# The Role of the Hedgehog Pathway in Cholangiocarcinoma

**DOI:** 10.3390/cancers13194774

**Published:** 2021-09-24

**Authors:** Giulia Anichini, Laura Carrassa, Barbara Stecca, Fabio Marra, Chiara Raggi

**Affiliations:** 1 Core Research Laboratory, Institute for Cancer Research and Prevention (ISPRO), 50139 Florence, Italy; giulia.anichini@gmail.com (G.A.); laura.carrassa@gmail.com (L.C.); b.stecca@ispro.toscana.it (B.S.); 2 Department of Experimental and Clinical Medicine, University of Florence, 50139 Florence, Italy

**Keywords:** biliary tract cancer, non-canonical, oncogene, targeted therapy

## Abstract

**Simple Summary:**

Cholangiocarcinoma (CCA) is one of the most refractory malignancies with a high mortality rate. Among all the pathways involved in CCA development, emerging evidence highlights Hedgehog (HH) signaling as a substantial player in CCA-genesis and development. The pro-tumoral function of HH provides potential therapeutic implications, and recently the use of HH inhibitors has paved the way for clinical application in various solid tumors. Targeting HH members, namely Hedgehog ligands, SMO transmembrane protein and GLI transcription factors may thus confer therapeutic options for the improvement of CCA treatment outcome.

**Abstract:**

Cholangiocarcinoma (CCA) is a poorly treatable type of cancer and, along with hepatocellular carcinoma (HCC), is the predominant type of primitive liver cancer in adults. The lack of understanding of CCA biology has slowed down the identification of novel targets and the development of effective treatments. While tumors share some general characteristics, detailed knowledge of specific features is essential for the development of effectively tailored therapeutic approaches. The Hedgehog (HH) signaling cascade regulates stemness biology, embryonal development, tissue homeostasis, and cell proliferation and differentiation. Its aberrant activation has been associated with a variety of solid and hematological human malignancies. Several HH-inhibiting compounds have been indeed developed as potential anticancer agents in different types of tumors, with Smoothened and GLI inhibitors showing the most promising results. Beside its well-established function in other tumors, findings regarding the HH signaling in CCA are still controversial. Here we will give an overview of the most important clinical and molecular features of cholangiocarcinoma, and we will discuss the available evidence of the crosstalk between the HH signaling pathway and the cholangiocarcinoma cell biology.

## 1. Introduction

Primary liver cancer (PLC) is one of the most common cancers worldwide and is the second leading cause of cancer-related mortality [1,2]. Primary liver tumors are grossly classified in hepatocellular carcinoma (HCC) and cholangiocarcinoma (CCA). HCC accounts for approximately 90% of all PLC, while CCA, a rare tumor but with an increasing global incidence, is the second most common form and accounts for about 5% of all PLC [1,2]. CCA is a highly heterogeneous disease arising from neoplastic transformation of intra- and extra-hepatic biliary epithelial cells (cholangiocytes), and it is characterized by a very poor prognosis [3,4].

The high mortality rate of CCA may depend on its nonspecific silent clinical features, which make it difficult to diagnose [5]. Currently, diagnosis is based on a combination of modalities, but, so far, no specific markers have been identified [1,2].

Another aspect contributing to the very poor survival rate of this tumor is its unresponsiveness to conventional therapies [3,4]. Currently, the standard-of-care treatment for CCA is limited to surgical resection, with 5-year survival of 20 to 40% [1,2]. Unfortunately, CCAs are generally asymptomatic in early stages and are usually diagnosed at an advanced unresectable stage and, although chemotherapy improves the quality of life of these patients, it remains only a palliative treatment [1,2]. Most patients with unresectable CCA undergo a rapid decline in clinical condition and die within 12 months of the onset of symptoms. To improve the outlook for individuals with CCA, both clinical and bench science are therefore imperative.

The goal of this review is to highlight the importance of the Hedgehog signaling pathway in the cholangio-carcinogenesis as a novel druggable candidate for this disease.

## 2. Cholangiocarcinoma Overview

Cholangiocarcinoma (CCA), a heterogeneous group of malignancies, occurs at any location along the biliary tree. It is anatomically classified as intrahepatic (iCCA), perihilar (pCCA), and distal extrahepatic (dCCA) [1,2,6,7]. On the other hand, iCCA can also be morphologically subcategorized into mass-forming (MF-iCCA), periductular-infiltrating (PI-iCCA), or intraductular growing (IG-iCCA). The MF-iCCA represents the most frequent form, whereas IG-iCCA is the least common form but with a more favorable prognosis [1].

During the past two decades, the incidence of iCCA as well as its mortality rate has been increasing worldwide, leading to the development of a growing scientific interest for this dismal malignancy. By contrast, the rate of eCCA is stable or even decreasing [1].

iCCA and eCCA, besides showing opposite epidemiological behaviors, are associated with different risk factors and histological features; diverse clinical outcomes; and dissimilar background in terms of expression profiling, pattern of genetic mutations, and epigenetic changes [1,6,7,8,9].

Some of the most prevalent CCA genetic alterations affect well-known cancer drivers such as tumor suppressor TP53, which regulates cell cycle, and KRAS, a tyrosine kinase signaling member [1]. About 20% of CCA showed TP53 mutation, whereas 7–54% of both iCCA and eCCA retained activating KRAS mutations [1,10]. Mutations in both genes were described as early events for CCA onset [1]. Moreover, 22% of iCCA presented proto-oncogene BRAF mutations, while inactivation of SMAD4 were described in 35% of iCCAs and 50% of eCCAs. Indeed, it has been hypothesized that there is a tumor suppressive role of SMAD4 by cell cycle regulation and the TGF-β pathway [1]. Taken together, these data indicate that genomic instability, TGF-β signaling, and RAS/RAF pathways may be driver for CCA onset.

Recent studies of next generation sequencing (NGS) have highlighted a highly mutated group of genes, including MLL3, RNF43, PEG3, ROBO2, BAP1, ARID1A, PBRM1, IDH1, and IDH2 [1,11], which are involved in histone modifier deactivation, G-proteins activation, and gain of genomic instability [11]. Notably, IDH1 and IDH2 mutations occurred together with an increased TP53 level [7]. Conversely, most of the tumors with mutations in the H3K4-specific methyltransferase MLL3 do not harbor TP53, KRAS, or SMAD4 mutations [1]. Interestingly, PEG3 and RNF43 are ubiquitin ligases and regulators of TP53 with the role of suppressing p53-mediated apoptosis. Moreover, RNF43 is even involved in the Wnt signaling pathway, targeting frizzled receptors for degradation. In Ov-related iCCAs, for example, it has been demonstrated as an upregulation of Wnt3a, Wnt5a, and Wnt7b [1]. ARID1A, PBRM1, and BAP1 together constitute the SWI/SNF complex, which is involved in nucleosome remodeling, mediating ATP-dependent chromatin remodeling processes. These tumor suppressor genes are frequently mutated and consequently silenced in CCAs [1].

Several observations underline how genetic variations may change according to etiology and CCA anatomical locations. BAP1 and IDH are more frequently mutated in non-liver–fluke-related CCAs, whereas TP53 mutations occur with increased frequency in liver–fluke-related tumors [10]. Moreover, although IDH1/2 and BRAF mutations are significantly predominant in the intrahepatic form of CCA, KRAS and TP53 mutations are relatively common in all CCA subtypes [1,6].

Others key mediators of cholangiocarcinogenesis are COX-2, MET, interleukin IL-6, and iNOS, as well as Notch, Hedgehog, and ErbB receptor kinase family members. In CCA, the soluble ligand EGF operates through the EGF receptor (EGFR), the founding member of the ErbB family, and increase CCA growth [1]. EGFR is deregulated in 32% of CCA patients, including both iCCA (11–27%) and eCCA (5–19%) [1,11]. Mutations in the tyrosine kinase domain of EGFR correlate with increased phosphorylation of AKT or p42/44 MAPK and p38 MAPK [1,12]. Human epidermal growth factor receptor 2 (ERBB2) is another member of the EGFR family responsible for CCA progression [11].

MAPK cascade can be activated even by MET, the receptor of hepatocyte growth factor (HGF), or IL-6, which represents a key cytokine in the CCA pathogenesis [11]. The binding of HGF to MET activates several downstream signaling pathways (RAS/MAPK, PI3K/AKT, and JAK/STAT) that drive tumor aggressiveness. MET over-expression has been described in both iCCA (20–60%) and eCCA (0–70%) [1]. In addition to the activation of MAPK cascade, IL-6 mediates the overexpression of EGFR as well as the STAT3-dependent upregulation of antiapoptotic protein MCL-1 [1].

In addition to intra-tumoral heterogeneity due to genetic alterations, heritable epigenetic alterations also generate phenotypical variability within tumors. Indeed, several epigenetic alterations, such as promoter hypermethylation and microRNA (miRNA) dysregulation, have also been linked to CCA development. Specifically, IDH mutations are associated with DNA hypermethylation of CpG shores, which suggests a global transcriptional deregulation. Importantly, the resulting deregulation of HNF4α blocks hepatocytic differentiation and promotes bile duct cancer development [1,13]. Moreover, in CCA, hypermethylated promoters silence tumor suppressor genes such as CDKN2A or p16INK4A, SOCS3, RASSF1A, and APC [11]. In parallel miRNAs also play an important role in CCA onset. For example, over-expressed miR21 has tumorigenic effects, by inhibiting programmed cell death 4 and tissue inhibitor of matrix metalloproteinase 3. Moreover, miR21 is able to regulate PI3K signaling, affecting CCA chemosensitivity [7,11].

Few studies have assessed the roles of chromosome aberrations such as copy number variations and gene fusions in the pathogenesis of human CCA. Notably, a study of copy number variation iCCA (*n* = 149) samples showed a range of chromosomal alterations, comprising gains at 1q and 7p and losses at 3p, 4q, 6q, 9pq, 13q, 14q, 8p, 17p, and 21q [7].

The advent of NGS approaches has further enriched the genomic landscape of CCA, reinforcing our understanding regarding its pathogenetic processes. Recent studies show involvement of fibroblast growth factor receptor 2 (FGFR2) and ROS proto-oncogene 1 (ROS1) fusions, together with an FGFR2–periphilin 1 (PPHLN1) fusion product. FGFR2 (chromosome 10q26) is one of the members of the fibroblast growth factor receptor family (FGFR1-4). FGFR2 alterations have primarily been detected in 6–50% of iCCA and 0–5% of eCCA, thus representing a putative critical diagnostic marker, as these rearrangements are identified almost exclusively in iCCA. FGFR2 fusions are the product of the combination of FGFR2 (exons 1–19) with disparate partners (i.e., BICC1, AHCYL1, MGEA5, TACC3, KCTD1, and TXLNA) [1]. The resulting fusion protein is activated through enforced dimerization, followed by the phosphorylation of the intracellular tyrosine kinase domain and the activation of downstream signaling pathways, including JAK/STAT pathways, PIK3/AKT/mTOR, and MAPKs [1]. Remarkably, a significant correlation has been observed between the presence of FGFR2–PPHLN1 and FGFR2–BICC1 fusion genes and the occurrence of mutations in KRAS genes, suggesting a possible cooperative role in coordinating iCCA pathogenesis [6]. FGFR2 fusions might thus represent the most prevailing targetable alteration in CCA; however, the sensitivity of the different FGFR2 fusion proteins to specific FGFR2 inhibitors remains undefined and should be broadly investigated in future studies.

Furthermore, 8.7% of CCA patients retained the FIG-ROS fusion gene that activates downstream signaling molecules, including STAT3 and AKT [1], thus accelerating tumorigenesis in a mouse model of iCCA holding KRAS and TP53 mutations [6].

Notably integrative genomic analysis identified two major iCCA types: inflammatory (STAT3 activation; cytokeratins overexpression) and proliferative (RAS, MAPK, and c-MET activation; KRAS and BRAF mutations) [1,9].

All these studies highlight the CCA heterogeneity which mirrors the underlying variability in molecular profile, drug-response, and outcome.

## 3. Current Therapeutic Intervention in Cholangiocarcinoma

### 3.1. Surgery

To date, radical surgical resection is the preferred therapeutic option for CCA, offering a curative chance. However, because of the challenging anatomical location and of the clinically “silencing” of this malignancy until later stages, only approximately 35% of patients with CCA undergo surgery [14]. Moreover, bilateral, multifocal disease, metastases, high post-surgical recurrence rates (generally 1–2 years) as well as comorbidities related to surgical risk, effectively decrease the real expected surgical benefits [15]. Frequent post-surgical relapse led to an implementation of several studies of adjuvant therapy. The BilCap study showed increased overall survival (OS) after surgical resection followed by Capecitabine treatment (51 months in the treated arm vs. 36 months in the observation arm) in iCCA. On the other hand, the resection of biliary tract cancer followed by adjuvant chemotherapy with gemcitabine and oxaliplatin (GEMOX), three months later, did not increase recurrence-free survival of CCA patients (PRODIGE12 study) [16]. Recently, the liver transplantation option has been considered in some specific disease conditions. For example, liver transplantation in unresectable pCCA preceded by neoadjuvant chemoradiotherapy showed admirable long-term disease-free survival rates [17,18]. Recently it has been demonstrated that very early iCCA patients after liver transplantation present good oncologic and survival outcome, although more confirmations are needed, such as the clarification of the systemic neoadjuvant regimens to be associated [19,20].

### 3.2. Systemic Chemotherapy

Patients with advanced or unresectable CCA require systemic chemotherapies as curative opportunity, although they have limited effectiveness and they are mostly palliative. The current standard of care is gemcitabine coupled with cisplatin [21]. The ABC-02 Phase III trial, which randomized 410 patients to gemcitabine alone or cisplatin/gemcitabine, demonstrated that cisplatin/gemcitabine, compared with gemcitabine monotherapy, prolongs OS by 3.6 months (11.7 months vs. 8.1 months, respectively) [22]. First-line treatment agents are associated with several side effects such as nausea, vomiting, and anorexia, and often fall into the development of chemoresistance. Recently, large randomization trials elucidated the possible benefits of second-line treatments. The phase III ABC-06 clinical trial randomly assigned 162 patients with advanced biliary tract cancer (72% CCA) progressing on first-line cisplatin-gemcitabine to either active symptom control (81 patients) or active symptom control with FOLFOX (folinic acid, 5-FU, and oxaliplatin) second-line chemotherapy (81 patients) [23]. Results of the trial showed a modest 0.9-month OS benefit in patients treated with FOLFOX, and clinically meaningful differences in survival at 6 and 12 months, suggesting that FOLFOX can be considered a good candidate in the second-line setting. Recently, results from a phase II clinical trial showed that treatment with nab-paclitaxel plus gemcitabine-cisplatin provided advantages in terms of median progression-free survival and overall survival as compared to controls treated with gemcitabine-cisplatin alone [24]. The gemcitabine-cisplatin-nab paclitaxel is now being compared in phase III setting to gem/cis as a new first-line standard (SWOG clinical trial number S1815).

### 3.3. Other Specific in Loco Therapies

Locoregional therapies based on the focalized delivery of chemotherapy and radiotherapy are also considered valid options for patients with localized unresectable CCA [25,26]. Increased survival and improved local control in locally advanced-metastatic iCCAs have been achieved with the following techniques: transarterial chemoembolization, transarterial radioembolization, hepatic arterial-based therapies, radiofrequency ablation, and photodynamic therapy. However, recurrence rates are still elevated [27].

### 3.4. Molecular Targeted Therapy

The high genetic variability of CCA represents a major challenge for effective pharmacological treatment; indeed, currently it remains one of the most dismal tumors with very limited therapeutic options. In recent years, deeper genomic studies and the advent of the next generation sequencing showed that nearly 45% of patients present potentially targetable genetic alterations, opening the possibility for new therapeutic opportunities [28,29]. The recent results of the MOSCATO-01 trial, which represent the first large-scale evaluation of precision medicine in hard-to-treat cancers, further support this evidence. In the trial, 68% of the comprised 43 biliary tract cancer (BTC) cases (29 iCCA, 10 eCCA, and 4 gallbladder cancer) showed actionable alterations, making this tumor one of the most potentially targetable malignancies [30,31]. The proper targeted therapies increased progression-free survival (PFS) in BTC patients as compared to those patients that could not be included in such targeted therapy trials. Thus, precision medicine seems to really have a good potential to improve BTC therapy. Indeed, the high requirement of introducing new therapeutic strategies in cholangiocarcinoma is completely revolutionizing the standard treatment algorithms for this disease, and many specific therapies are entering clinical practice in cholangiocarcinoma. The most significant targeted therapies associated with cholangiocarcinoma actionable molecular alterations currently in clinical trial development are briefly described below.

### 3.5. FGFR Inhibitors

Approximately 15–28% of patients with iCCA have tumors with FGFR2 rearrangements [28,29,32]. Pemigatinib (Pemazyre), a reversible adenosine triphosphate–competitive FGFR kinase inhibitor, was FDA-approved in 2020 as a targeted therapy for cholangiocarcinoma based on the results of the FIGHT-202 trial (NCT02924376) [33]. In this trial, 107 patients with CCA harboring FGFR2 rearrangements who were previously treated with chemotherapy were treated with pemigatinib once daily. An impressive 36% objective response rate and a median PFS of 6.9 months was observed. Futibatinib, an irreversible FGFR inhibitor also showed impressive activity in FGFR2-rearranged iCCA (NCT02052778) [34]. Importantly, futibatinib has been shown to overcome acquired resistance to reversible FGFR inhibitors, thus opening the potential to offer prolonged benefit by sequential FGFR inhibitor therapy [35]. Very recently, FDA granted the accelerated approval to Infigratinib for adults with previously treated, unresectable locally advanced or metastatic CCA with a fibroblast growth factor receptor 2 (FGFR2) fusion or other rearrangement [36]. Some studies with FGFR inhibitors (i.e., the FIGHT-302 study (NCT03656536) and the PROOF study (NCT03773302) are currently being considered in first-line setting [1,37].

### 3.6. IDH1/2 Inhibitors

Pathogenic mutations in isocitrate dehydrogense IDH1 and 2, a metabolic enzyme involved in the conversion of isocitrate to α-ketoglutarate, are among the most recurrent mutations, occurring in approximately 25% of iCCA patients [28,38,39]. Many IDH-selective inhibitors have been developed for cholangiocarcinoma patients in the last year, with AG-120 (ivosidenib, Agios) being the most evolved. This is an oral IDH1 inhibitor, FDA approved for the treatment of IDH1-mutated acute myeloid leukemia and very recently for advanced or metastatic CCA. In the phase III trial ClarIDHy (NCT02989857), 185 patients (91% IHCC) with IDH1-mutated cholangiocarcinoma who had received 1 or 2 lines of prior therapy were treated with either ivosidenib or placebo. Results showed a similar objective response rate (2.4% vs. 0%, with ivosidenib and placebo, respectively) but a modest benefit in PFS (2.7 months compared with 1.4 months for patients treated with placebo) and limited adverse toxicities [40,41]. Recently emerging combination strategies with IDH1 inhibitors are being tested in clinical trials with the intent to improve benefits, limiting toxicities, and possibly overcoming acquired resistance mechanisms. Based on recent preclinical evidence that IDH1-mutated malignancies present alteration in homologous recombination pathway and an increase in PARP inhibitor sensitivity, some preclinical studies and clinical trials investigations are undergoing in IDH mutated-CCA testing the drug combination between IDH and PARP inhibitors [42,43,44].

Moreover, a combination of IDH inhibitors with systemic chemotherapy or immunotherapy is another evaluable therapeutic opportunity [39].

### 3.7. BRAF-Directed Therapy

BRAF V600E mutations are present in approximately 3% of iCCA [28,29]. A single-arm, multicenter phase 2 ROAR trial (NCT02034110) elucidated the activity of the BRAF/MEK inhibitor combination in the context of CCA by evaluating the BRAF inhibitor dabrafenib (Tafinlar) combined with the MEK inhibitor trametinib (Mekinist) [45]. Results showed that the combination had a 47% objective response in patients with BRAF V600E–mutated cholangiocarcinoma. Patients with CCA enrolled in this trial had a median PFS of 9.2 months and median OS of 11.7 months [45].

### 3.8. Other Molecular Targets

Several other targetable molecular abnormalities occur in CCA. Notably, many reports highlighted the presence of cell cycle dysregulations and abnormalities, DNA damage response (DDR) pathway deficiency, and genomic instability [46,47]. Mutations in the most studied DDR genes BRCA1/2 fluctuates from 1% to 7% in patients with CCA; thus, after their success in pancreatic cancer, a plethora of studies aimed at investigating the response of CCA patients to PARP inhibitors, both in monotherapy and in combination with chemo and immunotherapy, are ongoing [43,48]. Other targetable DDR and cell cycle regulators of potential interest for CCA are being evaluated prevalently in preclinical setting (i.e., CDK4/6, Wee1, and ATR) [49,50,51]. Nonetheless, mutations in the epigenetic factors BAP1 and ARID1A are also emerging in CCA as potential actionable targets in synthetic lethality with PARP inhibitors and agents inducing DSB [28,52,53]. Other translocations such as ALK, ROS1, and NTRK and HER2 amplifications and mutations are present at low frequencies in CCA [28,54,55]. In summary, a plethora of targetable molecular pathways involved in CCA biology may contribute to ameliorate CCA therapeutic options. The Hedgehog pathway is among these pathways, but its role and mechanisms of action in this disease are still not fully understood [56,57].

## 4. The Hedgehog Signaling Pathway

The Hedgehog (HH) signaling cascade was first discovered as a key morphogenetic pathway in driving the embryonal development of fruit fly larva [58]. Its function is highly conserved in mammals, where it crucially participates in the regulation of essential cellular processes, including cell proliferation and differentiation, stemness, metabolism, tissue regeneration, and homeostasis [59,60]. Being fundamental in controlling such processes, the dysregulation of the HH cascade has been associated with diverse developmental disorders and cancer diseases [61].

A Hedgehog ligand is required to initiate the intracellular signaling, which culminates with the activation of the final effectors of the entire cascade, which are the GLI transcription factors. In mammals, three different Hedgehog ligands stimulate Hh-responsive cells. All three operate as morphogen proteins in regulating different aspects of the embryonal development, and they share comparable physiological effects, but with a different expression pattern and differential cellular responses, depending on ligand concentration [61,62,63]. Sonic Hedgehog (Shh) is the predominantly expressed Hh ligand in mammals [64,65], with Indian Hedgehog (Ihh) being partially redundant with Shh [61,66,67,68] and Desert Hedgehog (Dhh) mostly limited to the gonads, where its signal is important for Sertoli cells and granulosa cells [69,70,71].

In the absence of any Hh ligand, the 12-pass transmembrane receptor Patched 1 (PTCH1) occupies the base of the primary cilium, a specialized organelle required for the Hedgehog machinery, hindering the activity of the main HH transducer Smoothened (SMO) [72,73]. PKA, CK1, and GSK3β kinases phosphorylate GLI2 and GLI3, which are subsequently recognized by the F-box protein β-transducing-repeat-containing protein (β-TrCP) and sequestered outside the nucleus through a physical interaction with Suppressor of Fused (SuFu) [74,75,76]. Within the cytoplasm, GLI2 and GLI3 undergo proteasomal partial cleavage, reverting to their C-terminally truncated repressor forms (GLI^R^) and suppressing the transcription of HH target genes [77]. Conversely, GLI1 is predominantly regulated at the transcriptional level and through the ubiquitin-proteasome system (UPS)-dependent recognition of two degradation signals, which are degron DC and degron DN, located at the GLI1 C-terminus and the N-terminus, respectively [78].

**Figure 1:**
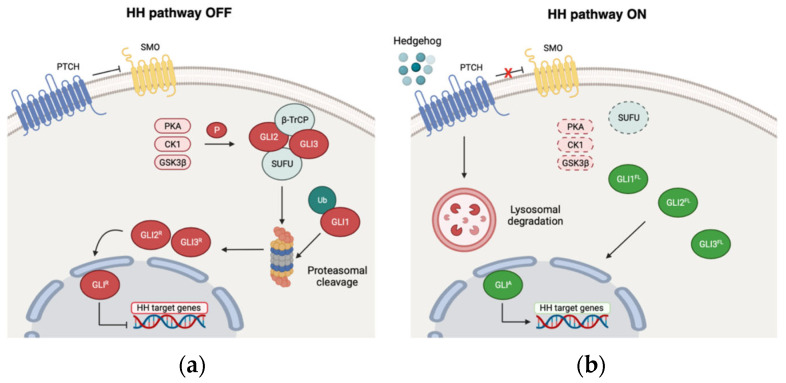
The canonical Hedgehog-GLI signaling. In the absence of any Hedgehog ligand stimulation (**a**), the transmembrane receptor PTCH1 inhibits the G-coupled protein Smoothened (SMO). GLI transcription factors are phosphorylated by PKA, CK1, and GSK3β and are thus recognized by SuFu, which sequesters them outside the nucleus. Herein, GLI2 and GLI3 are processed into the repressive forms, which hinder the transcription of Hedgehog target genes. GLI1 is degraded through the ubiquitin-proteasome system (UPS). The presence of a Hedgehog ligand that specifically binds PTCH1 (**b**) releases the inhibitory regulation of SMO. Accordingly, SMO initiates an intracellular cascade, which terminates with the translocation of the GLI as active forms into the nucleus, promoting the transcription of Hedgehog target genes.

Once secreted, the Hh ligand specifically binds the 12-pass transmembrane receptor PTCH1, which is therefore rapidly internalized and degraded [72,73]. The 7-pass transmembrane G-coupled receptor Smoothened is now released from PTCH1 inhibitory activity and translocated into the tip of the primary cilium, being therefore phosphorylated and activated by CK1α and GPCR kinase 2. SMO initiates an intracellular cascade, which prevents GLI suppressive modifications, promoting the migration of full-length GLI1, GLI2, and GLI3 into the nucleus as active forms (GLI^A^), in order to stimulate the transcription of HH target genes [79,80,81]. Among them, there are several factors implicated in controlling cell proliferation, survival, invasiveness, and stemness, together with GLI1 and PTCH1, thus creating a feedback loop, which further modulates the entire cascade itself [82,83] (Figure 1).

Beside the canonical process that turns on the Hedgehog signaling, different mechanisms are responsible for an aberrant activation of the cascade in cancer cells. Genetic alterations affecting HH fundamental members, i.e., loss of function (LOF) mutations in PTCH1, gain of function (GOF) mutations in SMO, and amplifications of GLI transcription factors, as well as dysregulated ligand-dependent stimulations (autocrine, paracrine, reverse paracrine), can be responsible for a non-canonical induction of the cascade [83,84,85,86,87]. In addition, an intricate network of intracellular interactions with other oncogenic pathways enables to bypass the entire HH upstream regulation by directly modulating the function of GLI transcription factors, both at a transcriptional and a post-translational level. Hence, a cooperative integration with RAS/RAF/MEK/ERK, PI3K/AKT, TGF-β cascades, among others, promotes the uncontrolled pro-tumoral activity of the GLI transcription factors in different types of human malignancies, including those of skin and lung, and also brain, gastrointestinal, and haematological tumors [59,88,89,90,91,92].

### 4.1. The Hedgehog Signaling in the Liver

The HH signaling remains mainly quiescent during adult life, where its activity is required for tissue regeneration. Therefore, as mentioned, an aberrant reactivation of the pathway has been found to be responsible for cancer initiation and progression in a variety of both solid and hematological human malignancies [53,93,94,95,96].

Several studies suggest that the Hedgehog cascade plays major roles in regulating both embryonal development and adult repair of the liver [97,98,99,100,101]. Namely, in the adult hepatic physiology, the HH signaling is nearly dormant, due to poor production and secretion of Hedgehog ligands by liver cells [102,103,104] and, on the other hand, to strong expression of Hedgehog inhibitors (i.e., Hhip) by liver sinusoidal cells, such as endothelial cells and quiescent hepatic stellate cells (HSCs) [105,106,107,108]. Furthermore, the HH signaling activity is gradually suppressed throughout the maturation and differentiation of liver epithelial cells [99].

Nonetheless, the HH signaling can be strongly reactivated during liver repair response upon the exposure to different types of injuries [98,100]. Hence, failing in proper regulation of the balance between latent/active HH cascade has been broadly associated with chronic liver disease and liver transformation [100,101,109,110].

Concerning liver tumors, an aberrant upregulation of the HH pathway has been demonstrated to be involved in both initiation and progression of HCC, hepatoblastoma, gallbladder cancer, and CCA [57,111,112,113,114,115,116,117,118].

### 4.2. Hedgehog and Cholangiocarcinoma

Being cholangiocarcinoma the second most frequent type of liver cancer, with very limited available therapeutic strategies and poor outcomes, the necessity to delve into understanding the molecular landscape of this biliary malignancy becomes explicit. Even though the function and the mechanisms of action of the HH pathway in CCA biology are still poorly explored and not completely understood, available data suggest a prominent role for HH in supporting cholangiocarcinoma malignant properties.

#### 4.2.1. Hedgehog in CCA Patients

As mentioned before, one of the mechanisms responsible for driving an aberrant HH signaling in tumor cells concerns genetic alterations involving HH pathway key components. Both mutation and copy number alterations (CNA) affecting Hedgehog pathway main members, namely GLI1, GLI2, GLI3, SMO, PTCH1/2, SUFU, and DHH, have been observed in mixed cholangiocarcinoma patients with different frequencies (Table 1) [119,120]. GLI3 is one of the most frequently mutated genes (4.6%), together with Desert Hedgehog (5.6%), whose function in human cancer is still poorly investigated. Nonetheless, DHH expression has been observed in gastric cancer [121], correlates with advanced tumor grades in breast cancer [122], and has been supposed as a prognostic indicator for clear cell renal carcinoma [123] and for a specific subgroup of pediatric AML [124]. By the way, the biological significance of those HH alterations identified in CCA patients is still not characterized. Amplifications detected in GLI1 and DHH may assume gain of function effects, whereas, for missense mutations, we can only hypothesize inactivating alterations for the negative regulators PTCH1, PTCH2, and SUFU on one hand, and activating alterations for GLI transcription factors, SMO and Hedgehog ligands, on the other, as occurs in other types of cancer [85,125,126,127,128,129,130,131,132].

The expression of Hedgehog signaling members has been analyzed in a cohort of 50 human CCA tissues, where GLI1 and PTCH1 have been found overexpressed in approximately 50% and 30% of cases, respectively, together with SHH, detecting a significant activation of Hedgehog in almost 50% of cases [56]. Furthermore, a significant correlation of HH pathway activation with tumor progression and prognosis has been documented in CCA. GLI1 overexpression has been associated with malignant lymph node status, and SHH expression is enriched in undifferentiated tumors (grade 2 and 3), compared to highly differentiated tumors (grade 1) [133,134,135]. In a group of 200 patients with intrahepatic cholangiocarcinoma, SHH, IHH, PTCH1, GLI1, GLI2, and SMO have been observed overexpressed in more than 65% of clinical iCCA specimen, compared to non-malignant biliary epithelium, with SHH, SMO, and GLI2 being the most frequently upregulated HH members (>80%). Interestingly, in iCCA tissues, high GLI1 and/or GLI2 expression are significantly associated with intrahepatic metastasis and patient adverse disease-free-survival and overall survival [113,136], which has also been correlated with IHH and PTCH1 expression [112]. The Hedgehog cascade has been related also to CDH3 expression, which is associated with CCA progression to more aggressive stages and thereupon is correlated to limited overall survival [137]. Altogether, the reported studies provide a role for the Hedgehog-GLI signaling pathway, particularly for GLI1 and GLI2, as reliable prognostic factor for cholangiocarcinoma.

#### 4.2.2. Hedgehog Aberrant Activation in CCA Cells

Early evidence of HH pathway aberrant activation in BTC came from an analysis of mRNA expression in a panel of gastrointestinal cancer cell lines, including cholangiocarcinoma. Herein, SHH, IHH, GLI, and PTCH mRNA expression was widely detected [138]. GLI1, as in other tumors, was found to promote iCCA survival, growth, and EMT reprogramming [113,139], together with GLI3, which directly binds to the promoter of death receptor 4 (DR4), repressing its transcription and thus preventing TRAIL-dependent cholangiocarcinoma cell death [140]. Furthermore, the Hedgehog signaling was shown to stimulate the intracellular production of miR-25, a microRNA that is functionally involved in the protection of CCA cells towards TRAIL-determined cell death [141].

In CCA cell lines, the stimulation of the pathway with recombinant SHH ligand resulted as being very effective in activating HH-dependent transcriptional activity and in promoting in vitro cholangiocarcinoma cell growth, which were both negatively affected by the administration of the 5E1 Hedgehog-neutralizing antibody and by the genetic modulation of GLI1 [138]. The responsiveness of CCA cells to ligand stimulation could suggest a requirement for the canonical activation of HH in CCA cells. Nevertheless, cholangiocarcinoma cells often lack the ability to express cilia [57,142], which are cellular structures required for the transduction of the canonical pathway, and other studies have indeed provided evidences concerning a cilium and/or GLI-independent modality of HH activation in non-ciliated CCA cell lines [57,139]. The precise role of the primary cilium is currently thought to be context-dependent [143,144,145] and in cancer we can find a multitude of diverse aspects that can drive the non-canonical route. Thus, additional studies appear to be required, with the aim of elucidating the network of non-canonical regulation of Hedgehog in CCA cells.

Beside sustaining growth and proliferative processes, the HH pathway acts as a key mediator in coordinating many other cancer properties in a variety of human cancers, including survival, stemness, migration, invasion, deregulated metabolism, genome instability, angiogenesis, and pro-tumoral inflammation [146]. Hypoxia, which is an important element in contributing to malignant transformation and cancer progression, as well as chemoresistance [147,148,149], induces the activity of HIF-1α, which positively regulates the HH signaling with malignant outcomes in different types of human cancers [150,151], including cholangiocarcinoma, where hypoxia-induced HIF-1α promotes stemness features and invasive behavior by modulating SHH, SMO, and GLI1 [121]. HIF-1α appears to be preferentially expressed in CCA tissues, rather than in the neighboring normal biliary epithelium [152], and being such a relevant element in sustaining tumor chemoresistance and immune evasion, many hypoxia-targeting strategies are now under investigation for cancer therapy [153]. An accurate comprehension of the relationship between HH and hypoxia in CCA would thus be beneficial for the designing of powerful combinatorial targeted strategies.

Cholangiocarcinoma is characterized by a strong desmoplastic reaction, prompted by both fibroblastic and immunological cellular subsets, with HSC-derived myofibroblasts being one of the most predominantly represented stromal components. The intricate network that develops between tumor and the stromal counterpart appears to be relevant in sustaining malignant properties in cancer cells [154]. As many signaling molecules are released by stromal cells and then recognized by cancer cells, the CCA desmoplastic environment could represent an important factor in stimulating Hedgehog signaling in tumor cells in a paracrine way. Indeed, the suppressive effect of the HH signaling on TRAIL-induced cell death has been demonstrated to be mediated by myofibroblasts-secreted PDFG-BB, which signals in a paracrine manner in cancer cells, where it activates the Hedgehog cascade [155,156]. In this context, in the absence of any HH ligand stromal PDGF-BB induces the trafficking of SMO in a PKA-dependent manner, promoting the translocation of GLI2 into the nucleus and, ultimately, its transcriptional activity [157]. Once activated, the HH pathway protects CCA cells from TRAIL-induced apoptosis via, at least in part, a PLK2-mediated mechanism [126,127,155,156]. That Hedgehog-conditioned interplay between cholangiocarcinoma cells and the stromal counterpart is crucial, not only in preventing cancer cell death, but also in stimulating in vitro cancer cell proliferation, migration, and invasion [112]. In addition, the presence of a functional stromal microenvironment enhances tumor growth in vivo, along with activating the angiogenetic process in an HH-dependent manner and with making CCA cells more sensitive to SMO-antagonizing compounds [112]. This could be due to the fact that, beside cancer cells, the stromal cellular portion also displays an activation of the HH signaling, with Shh stimulating HSC-derived CAFs viability in an autocrine fashion [108]. A reciprocal HH-dependent regulation could thus be feasible in cholangiocarcinoma, as occurs in other types of gastrointestinal cancers [158,159]. Beside CAFs, CCA microenvironment results enriched different immunological cell subsets [154]. Among them, M2 macrophages have been demonstrated to sustain CCA growth in vivo in a WNT-dependent manner. Interestingly, the depletion of macrophages results in reduced CCA growth upon the downregulation of many signaling proteins, including SMO and PTCH1 [160], suggesting that this HH-conditioned interplay with CCA cells may involve not only CAFs, but also immunological cells.

#### 4.2.3. Hedgehog Targeting Strategies in CCA

Blocking the HH signaling by chemically targeting upstream or downstream HH mediators displayed promising effects in hindering CCA growth and progression. The currently available HH inhibiting compounds can be subdivided into 2 main groups: SMO and GLI inhibitors. The natural alkaloyd cyclopamine is the first discovered SMO inhibitor and has been largely used in CCA studies, providing early favorable data concerning the efficacy of targeting HH in this biliary tract neoplasia. Suppressing the activity of SMO with cyclopamine, alone or in combination with other appropriate targeted therapies, for instance MAPKs inhibitors [161], resulted as being strongly effective in repressing CCA growth and survival, but also in preventing cholangiocarcinoma invasion and metastasis [57,113,133,138,155]. Nevertheless, the emergence of limited oral solubility and serious side effects in mice restricts cyclopamine availability to pre-clinical studies and denotes the necessity to involve different HH-antagonizing compounds to select for putative clinical studies.

Vismodegib (GDC-0449) is a second generation cyclopamine derivative and the first SMO blocking agent to be FDA approved for clinical administration in advanced and metastatic BCC patients [162]. Vismodegib has also been found to be powerful as an anticancer agent in cholangiocarcinoma, reducing in vivo cholangiocarcinoma initiation, growth, and metastatic spreading [57], suggesting new potential strategies beyond cyclopamine to target the Hedgehog signaling in cholangiocarcinoma. Notwithstanding, the onset of mutations in the drug binding pocket of SMO that interrupt the responsiveness to vismodegib administration is frequent in BCC and MB patients [87,163,164]. Thus, despite the strong preclinical efficacy of targeting HH with cyclopamine derivatives in CCA, engaging novel SMO antagonizing structures that could offer more powerful mechanisms of target binding and biological action [165,166,167,168,169,170,171,172,173,174] should be properly considered for CCA studies.

**Figure 2:**
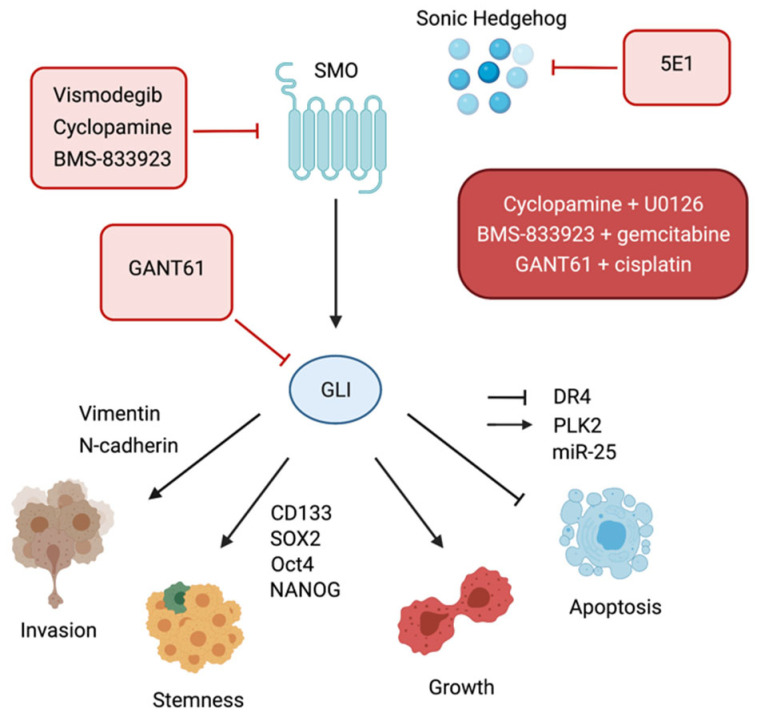
Schematic representation of the Hedgehog pathway and its functions in CCA. The HH signaling regulates growth, survival, stemness, and invasiveness in cholangiocarcinoma cells. HH pathway antagonists used in CCA preclinical studies are acting at the level of SHH (5E1 anti-SHH antibody), SMO (vismodegib, cyclopamine, BMS-833923), and the GLI (GANT61). They have been tested alone or in combination with other targeted molecules (U0126) or with chemotherapeutic drugs (gemcitabine, cisplatin) in vitro and in vivo. Proteins that are illustrated as HH downstream targets have been demonstrated to be modulated directly by transcriptional binding of GLI (DR4, PLK2) or as a final effect of the pharmacological inhibition of the HH cascade in CCA cells.

Hedgehog-antagonizing agents have also been demonstrated to be beneficial in combination with conventional chemotherapy to target biliary tract tumors. Intriguingly, the administration of the SMO inhibitor BMS-833923 in mouse xenograft models of cholangiocarcinoma potentiates the effects of the chemotherapeutic agent gemcitabine in reducing in vivo tumor volume [56]. Similarly, the combination of cisplatin with the GLI inhibitor GANT61 displayed synergistic effects in hampering in vitro cholangiocarcinoma cell growth and survival [134], providing interesting novel insights concerning the opportunity to target the non-canonical cascade by acting directly on GLI transcription factors, even in BTC (Figure 2). This therapeutic approach is attractive because, beside the outbreak of mutations in SMO, resistance to SMO inhibitors can be also driven by downstream compensatory mechanisms, which are responsible for the activation of the GLI (reviewed in [87]).

## 5. Concluding Remarks and Future Perspectives

HH signaling is an evolutionarily conserved pathway, being essential for coordinating normal development and differentiation in vertebrates. However, aberrant HH pathway activation is widely known to be closely correlated with the development and malignant progression of several human cancers. The available preclinical experimental data indicate that dysregulation of this pathway is a determinant of cholangiocarcinoma progression and metastasis (Figure 2). Nevertheless, in CCA, many aspects concerning the specific molecular mechanisms that drive the pro-tumoral function of the Hedgehog signaling still remain poorly understood. Trying to delve into the specific function of each member of the HH cascade, as well as comprehending the biological implications of related genetic alterations detected in CCA patients, thus becomes conceivable, in order to orientate the selection of HH targeting agents for further preclinical studies and hypothetical future clinical enrollment. Furthermore, there are many elements related to the crosstalk of HH signaling with other CCA-driving oncogenic pathways that are thus far open to question. In addition, in cancer, more than one non-canonical mode of activation can often be present, and canonical and non-canonical activation can coexist, shaping an intricate and sophisticated molecular landscape. Defining and elucidating the weight of this particular aspect of non-canonical activation, together with establishing the molecular mechanisms of action of HH targeting agents in CCA, could therefore become challenging, with the perspective of combining Hedgehog inhibitors with other targeted strategies, which appears to represent a promising system from available CCA preclinical studies. Because cholangiocarcinoma management is still grounded predominantly on systemic approaches, building up novel strategies based on blocking tumor-specific deregulated mediators should be seen as imperative. Furthermore, preclinical studies indicate that HH pathway inhibitors improve the efficacy of chemotherapeutic regimen. Lastly, a special consideration should be addressed to the profound interconnection between CCA cells and the stromal microenvironment, which not merely supports and sustains tumor progression, but also functions in affecting and restricting therapy responsiveness. For that reason, different strategies to target cancer microenvironments are currently under preclinical and clinical considerations in different human neoplasias [175], even though using TME-targeting strategies alone cannot reasonably ensure a complete eradication of the tumor. In CCA, some of the TME-driven mechanisms that promote malignant progression function in a Hedgehog-dependent manner; hence, a combinatorial targeting of HH and stromal function may be contemplated. In conclusion, this review aims to clarify the current knowledge of Hedgehog signaling in cholangiocarcinoma and highlights the possibility of promoting the study of innovative anti-CCA strategies based on HH-inhibiting approaches.

## Figures and Tables

**Table 1:** Genetic alterations affecting Hedgehog signaling pathway in CCA patients.

Gene	Missense	Nonsense	In/Del	Splicing	CNA	Frequency (%)
GLI1	Q867K, A108D, A941T, P821S, S89L, A670S, P842S, E1015D		G274Afs*6		AMP	2.5
GLI2	P386L	E431 *				2.7
GLI3	P575T, D848G, Y1223F, N1203K, R875C, S1025I, G1001S, E729K, L364I, G221=, R1010W, R220P, V778I, R1189H, R1010Q, G371D, P791L, A1005D, L690I, I254V, N1203K, D428N, T167M, E1014K, E551D	R667 *		X158_splice		4.6
SMO	S785L, G402V, T307I, A631S		L23dup			0.7
PTCH1	G880V, D301N, L775M, E405K, P1315L, A412T, D878H					1.1
PTCH2	A396V					2.9
SUFU	V323I, L68S					0.4
DHH	S384P				AMP	5.6

Data were obtained from cBioportal public databases. The frequency reported represents the sum of frequency in point mutations and copy number alterations. *: stop codon.

## Data Availability

Data sharing not applicable. No new data were created or analyzed in this work.
